# Facile methods for heterologous production of bacterial microcompartments in diverse host species

**DOI:** 10.1111/1751-7915.12869

**Published:** 2017-12-04

**Authors:** Todd O. Yeates, Thomas A. Bobik

**Affiliations:** ^1^ Department of Chemistry and Biochemistry University of California Los Angeles CA USA; ^2^ UCLA‐DOE Institute for Genomics and Proteomics Los Angeles CA USA; ^3^ Roy J. Carver Department of Biochemistry, Biophysics, and Molecular Biology Iowa State University Ames IA USA

Bacterial microcompartments, present in nearly 20% of known bacterial species, are giant proteinaceous structures consisting of a protein shell that encloses a series of sequentially acting metabolic enzymes (Bobik *et al*., 2015). Their general purpose is to prevent small, volatile or toxic pathway intermediates from being released into the cytosol. Depending on context, bacterial microcompartments are abbreviated in the literature alternatively as MCPs or BMCs; for clarity here (and for keeping with Graf *et al*.), we use MCP to describe a microcompartment, and we use the widely accepted name BMC for the family of proteins that comprise the main components of MCP shells. A few types of MCPs have been characterized experimentally, beginning first with the carboxysome (which enhances CO_2_ fixation in cyanobacteria and some chemoautotrophs), and then the Pdu MCP (which allows for the metabolism of 1,2‐propanediol without exposing the bacterial cytosol to the reactive intermediate propionaldehyde) (Fig. 1). More broadly, MCPs predicted to sequester diverse metabolic pathways have been identified by bioinformatic analyses and preliminary experimental studies (Abdul‐Rahman *et al*., 2013; Jorda *et al*., 2013; Axen *et al*., 2014). Structural studies have played a key role in showing that MCPs are not merely inclusions in bacterial cells, but highly sophisticated molecular machines satisfying the concept of true organelles in bacteria (Kerfeld *et al*., 2005; Yeates *et al*., 2011). Revelations about the complexity and sophistication of MCPs have spurred much research into understanding more fully how they evolved, how they operate and how they might be engineered for unique applications.

**Figure 1:**
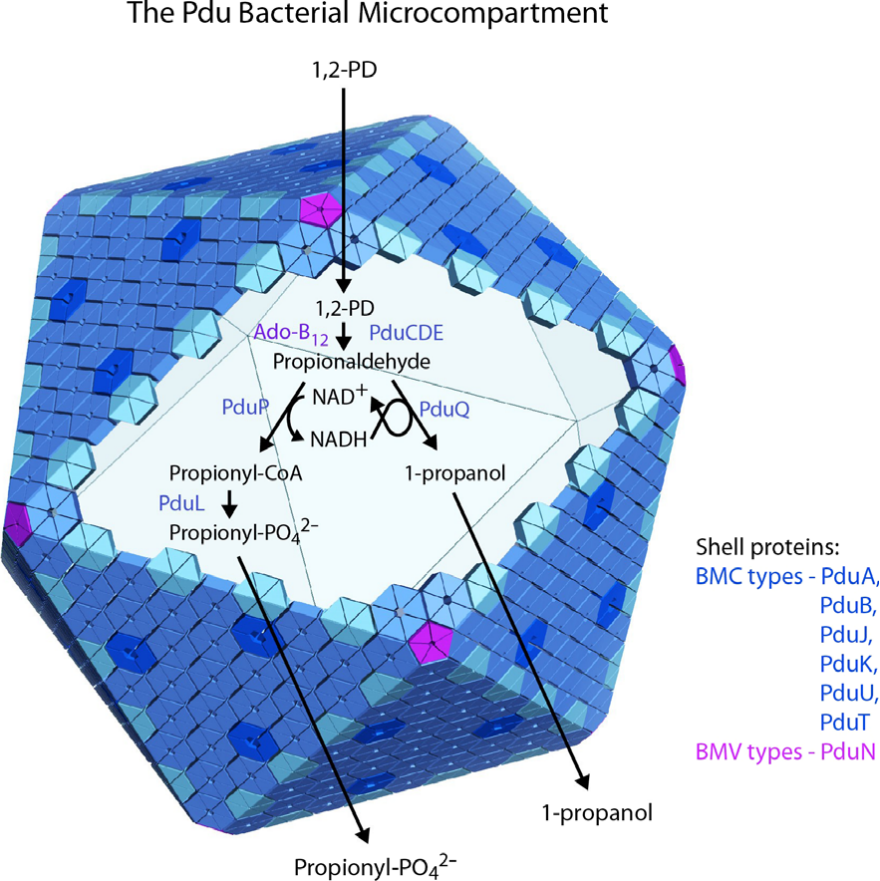
An idealized structural and metabolic model for the propanediol utilization (Pdu) bacterial microcompartment (MCP). A few thousand protein subunits, most belonging to the family of BMC shell proteins, encapsulate a series of enzymes for metabolizing 1,2‐propanediol (1,2‐PD) without allowing escape of the toxic aldehyde intermediate. Substrates and product pass selectively through pores in the shell proteins.

Within the broad scientific area of synthetic biology, diverse molecular systems are currently being pursued as potential frameworks for achieving spatial control over the various activities within cells (Lee *et al*., 2012). Among these, MCPs provide natural starting points for applications aimed at organizing metabolic processes (Kim and Tullman‐Ercek, 2013; Lawrence *et al*., 2014). They are especially attractive for applications where limited molecular exchange between different subcellular regions is desirable, either to improve channelling of substrates through multiple enzymatic reactions, or to limit the escape of toxic metabolites or even toxic proteins (Yung *et al*., 2017). In addition, owing to their size – since thousands of protein subunits comprise a single shell – MCPs have internal volumes large enough to encapsulate hundreds of enzyme molecules. This capacity offers an advantage over current strategies for designing novel enclosures by protein engineering; those efforts have so far produced designed protein assemblies with internal volumes one or two orders of magnitude smaller than MCPs (Bale *et al*., 2016; Yeates *et al*., 2016). On the other hand, the size and complexity of MCPs present numerous experimental challenges, e.g. for robust production and assembly in heterologous systems.

Several recent studies have advanced the goal of facile heterologous production of MCPs (Parsons *et al*., 2008; Bonacci *et al*., 2012; Sargent *et al*., 2013; Held *et al*., 2016). A common theme in many studies has been a reductionist approach to try to simplify or minimize the MCP shell by testing various subsets of shell proteins for their ability to assemble properly (Parsons *et al*., 2010; Choudhary *et al*., 2012; Lassila *et al*., 2014); some MCPs are comprised of seven or more different BMC shell proteins. The results have provided hints about the structural roles of individual shell proteins in MCP assembly, and have produced shells comprised of only a small number of distinct shell proteins, making them regular enough in shape to be structurally characterized (Jorda *et al*., 2016; Sutter *et al*., 2017).

New work described in this issue analyses the challenges of heterologous MCP expression from a different angle. Rather than investigating what types of MCP constructs – e.g. what subsets of natural shell genes – are compatible with assembly in a given target host (e.g. *Escherichia coli*), Graf *et al*. (2017) ask what hosts might be compatible with production of some MCP from its entire complement of genes. This is notable in view of the many genes required for native assembly and function of most MCP types (other than some carboxysomes, which are generally simpler). The authors focus on the Pdu MCP (Fig. 1) and the associated components of the *cob/cbi* cobalamin biosynthetic operon from *Salmonella* (cobalamin is required for 1,2‐propanediol metabolism inside the Pdu MCP). Including enzymes, structural components and regulatory proteins, this amounts to 43 genes across a 38 kb segment of DNA.

To execute their heterologous expression studies, Graf *et al*. (2017) used an FRT (flippase‐based) strategy for *in vivo* recombination to insert their 38 kb DNA segment into a plasmid with a broad host range across Gram negative bacteria. A plasmid harbouring all the *pdu* and *cob/cbi* genes allowed the investigators to rapidly assess the ability of diverse host bacterial species to produce native‐like MCPs. MCPs could be isolated from 6 of the 10 species tested. The differences in outcomes provide opportunities for future studies to dissect the origins of the genetic interactions observed, including host factors and regulatory mechanisms essential to MCP function and assembly. In addition, the successful production of MCPs in several new bacterial hosts will enable new functional studies and promote new MCP applications in diverse contexts.

## Conflict of interest

None declared.
